# LRP1 at the crossroads of Aβ clearance and therapeutic targeting in Alzheimer’s disease

**DOI:** 10.3389/fnagi.2025.1669405

**Published:** 2026-01-09

**Authors:** Yuepeng Deng, Haolin Yin, Zihao Lu, Huan Lan, Wenxiong Liu, Chao Zuo, Nanfang Pan, Xiaohe Tian, Qiyong Gong

**Affiliations:** 1 Department of Radiology, Huaxi MR Research Center (HMRRC), Institute of Radiology and Medical Imaging. West China Hospital of Sichuan University, Chengdu, Sichuan, China; 2 Psychoradiology Key Laboratory of Sichuan Province, West China Hospital of Sichuan University, Chengdu, Sichuan, China; 3 Xiamen Key Lab of Psychoradiology and Neuromodulation, Department of Radiology, West China Xiamen Hospital of Sichuan University, Xiamen, Fujian, China; 4 Research Unit of Psychoradiology, Chinese Academy of Medical Sciences, Chengdu, Sichuan, China

**Keywords:** Alzheimer’s disease, beta-amyloid, blood-brain barrier, low-density lipoprotein receptor-related protein 1, targeted therapy

## Abstract

Alzheimer’s disease (AD), characterized by progressive cognitive decline, memory impairment and behavioral disturbances, is the most common form of dementia, and no disease-modifying treatments are available to halt or slow its progression. Amyloid-beta (Aβ) is suggested to play a pivotal role in the pathogenesis of AD, and enhancing the clearance of Aβ from the brain has emerged as a major research direction. As the primary receptor for Aβ clearance at the blood-brain barrier (BBB), low-density lipoprotein receptor-related protein 1 (LRP1) plays a crucial role in regulating Aβ transport and metabolism. Understanding the mechanisms through which LRP1 functions, as well as the factors that influence its activity is essential for enhancing Aβ clearance from the brain and developing targeted therapeutic strategies for Alzheimer’s disease. In this review, we introduce the transport of Aβ across the BBB, followed by a discussion of the basic structure and function of LRP1 and its role in AD progression. Then, we summarize factors affecting LRP1 function and current advances in LRP1-targeted therapies. Finally, we explore the potential of LRP1 as a therapeutic target for AD. So, LRP1 may be a central modulator of Aβ dynamics and a clinically actionable target for treatment of Alzheimer’s disease.

## 1 Introduction

Alzheimer’s disease (AD), which is characterized by progressive cognitive decline, memory impairment and behavioral disturbances, is the most common form of dementia, accounting for 70–80% of all dementia cases (Jorm et al., 1987). AD can be classified into familial Alzheimer’s disease (FAD) and sporadic Alzheimer’s disease (SAD) based on genetic characteristics where FAD is primarily driven by mutations in genes such as *APP*, *PSEN1*, and *PSEN2,* while the pathogenesis of SAD involves complex interactions between genetic and environmental factors, with a significant influence from the pleiotropic effects of the *APOE ε4* allele (Blennow et al., 2006; Scheltens et al., 2021). Furthermore, AD exhibits phenotypic heterogeneity and can be categorized into early-onset AD (EOAD, onset age < 65 years) and late-onset AD (LOAD, onset age ≥ 65 years) in Clinical where EOAD is often associated with dominant genetic mutations, while LOAD is more influenced by metabolic dysfunction and vascular impairments (Reitz et al., 2020; Dorszewska et al., 2016).

Autopsy studies of AD patients revealed that amyloid plaques composed of β-amyloid (Aβ) peptides and neurofibrillary tangles (NFTs) formed by hyperphosphorylated tau protein are hallmark pathological features of the disease (Glenner and Wong, 1984; Kang et al., 1987; Grundke-Iqbal et al., 1986; Kosik et al., 1986; Kidd, 1963). Among the various hypotheses proposed for AD pathogenesis, the Aβ cascade hypothesis has dominated research for over three decades. According to this hypothesis, abnormal metabolism of amyloid precursor protein (APP) leads to excessive Aβ accumulation, which serves as a critical trigger in AD pathogenesis by inducing neuronal damage and neuroinflammation (Selkoe, 2008). In the brain, APP is a single-pass transmembrane protein concentrated in neuronal synapses, which is generated by brain neurons, blood vessels, blood cells, and a small number of astrocytes (Rice et al., 2019; O'Brien and Wong, 2011). Subsequently, APP is cleaved by two hydrolyses, including β-secretase extracellular and γ-secretase intracellular, to generate Aβ (Blennow et al., 2006). whereas in a diseased state, APP undergoes abnormal cleavage by β-secretase, resulting in the release of a shorter ectodomain, sAPPβ (N-terminal fragment), and retention of C99 (C-terminal fragment) in the membrane (Tiwari et al., 2019). C99 is then further cleaved by γ-secretase, releasing the APP intracellular domain, and the soluble Aβ peptides further polymerize to form aggregated plaques (Zhang et al., 2022). In the peripheral blood, platelets are the primary source of Aβ peptides and a large amount (more than 90%) of Aβ is derived from circulating platelets (Chen et al., 1995; Li et al., 1998). More than 90% of blood Aβ40 and over 97% of blood Aβ42 are related to plasma lipoproteins in human (Matsubara et al., 2004). However, the causality between the levels of peripheral blood Aβ and its aggregation in the brain, particularly the role of the peripheral blood Aβ in the pathology of AD, is still unclear (Shi et al., 2024). Aβ further accelerates disease progression by initiating a toxic cascade involving tau pathology and neurodegeneration (Busche and Hyman, 2020). However, a key contradiction that some individuals with Aβ deposition do not exhibit cognitive decline suggests that this hypothesis needs to integrate additional dimensions, such as tau pathology, neuroinflammation and metabolic imbalance to enhance its explanatory power (Olejar et al., 2024). Some researchers also propose that vascular dysfunction plays a crucial role in Aβ accumulation and neurodegeneration, particularly through the disruption of the blood-brain barrier (BBB) (Luissint et al., 2012).

**Figure 1:**
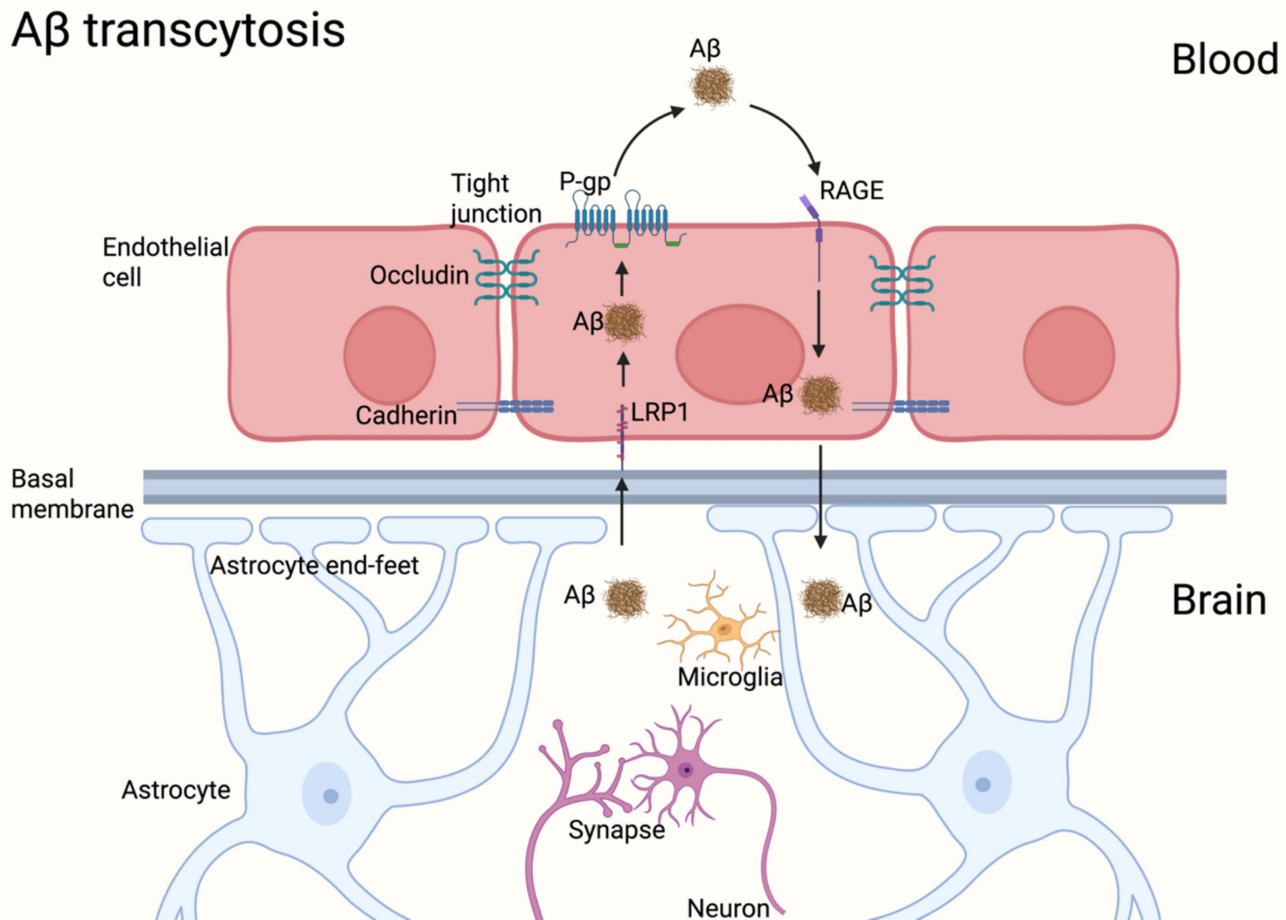
Structure and physiology of the BBB. BBB consists of cerebral endothelial cells, perivascular mural cells (pericytes), glial cells (astrocytes and microglia) and neurons, continuous tight junctions (e.g., occludin and claudin)1—and adherence junctions (cadherins) between brain endothelial cells. BBB regulates Aβ transport between the plasma and brain via RAGE and facilitates Aβ clearance through LRP1 and P-gp.(created by BioRender).

BBB represents a unique structure of the central nervous system (CNS), consisting of cerebral endothelial cells, perivascular mural cells (pericytes), glial cells (astrocytes and microglia) and neurons, continuous tight junctions (e.g., occludin and claudin)1—and adherence junctions (cadherins) between brain endothelial cells (Halliday et al., 2016; Davies et al., 2000; Sharabi et al., 2019). BBB regulates Aβ transport between the plasma and brain via the receptor for advanced glycation end products (RAGE) and facilitates Aβ clearance through cell surface receptors such as low-density lipoprotein receptor-related protein 1 (LRP1) and P-glycoprotein (P-gp) (Bell et al., 2007; Deane et al., 2003; Petrushanko et al., 2023). Studies indicate that the LRP1 is broadly expressed with clear and strong roles in neurons, astrocytes, and pericytes (Chen et al., 2022; Fan et al., 2024; Romeo et al., 2021). Moreover, the LRP1is expressed mainly at the abluminal side of the BBB, plays a pivotal role in maintaining Aβ homeostasis in the CNS (Bell et al., 2007; Deane et al., 2004; Shibata et al., 2000) (Figure 1).

Studies indicate that the BBB clearance dysfunction is a major contributor to age-related declines in Aβ transportation (Elbert et al., 2022). Aβ toxicity and metabolism have become central topics in AD research and therapeutic development because impaired Aβ clearance leads to its aggregation which is an initiating event in AD pathogenesis (Selkoe, 2006; Sun et al., 2018). Aβ clearance across the BBB primarily depends on receptor-mediated transport mechanisms, meaning that changes in receptor expression and function can directly impact Aβ clearance efficiency and lead to its accumulation. As LRP1 is the principal receptor involved in BBB-mediated Aβ clearance, investigating its expression levels and functional alterations is crucial for understanding AD pathogenesis. Additionally, targeting LRP1 for AD therapy holds great promise.

## 2 LRP1 structure and function

**Figure 2:**
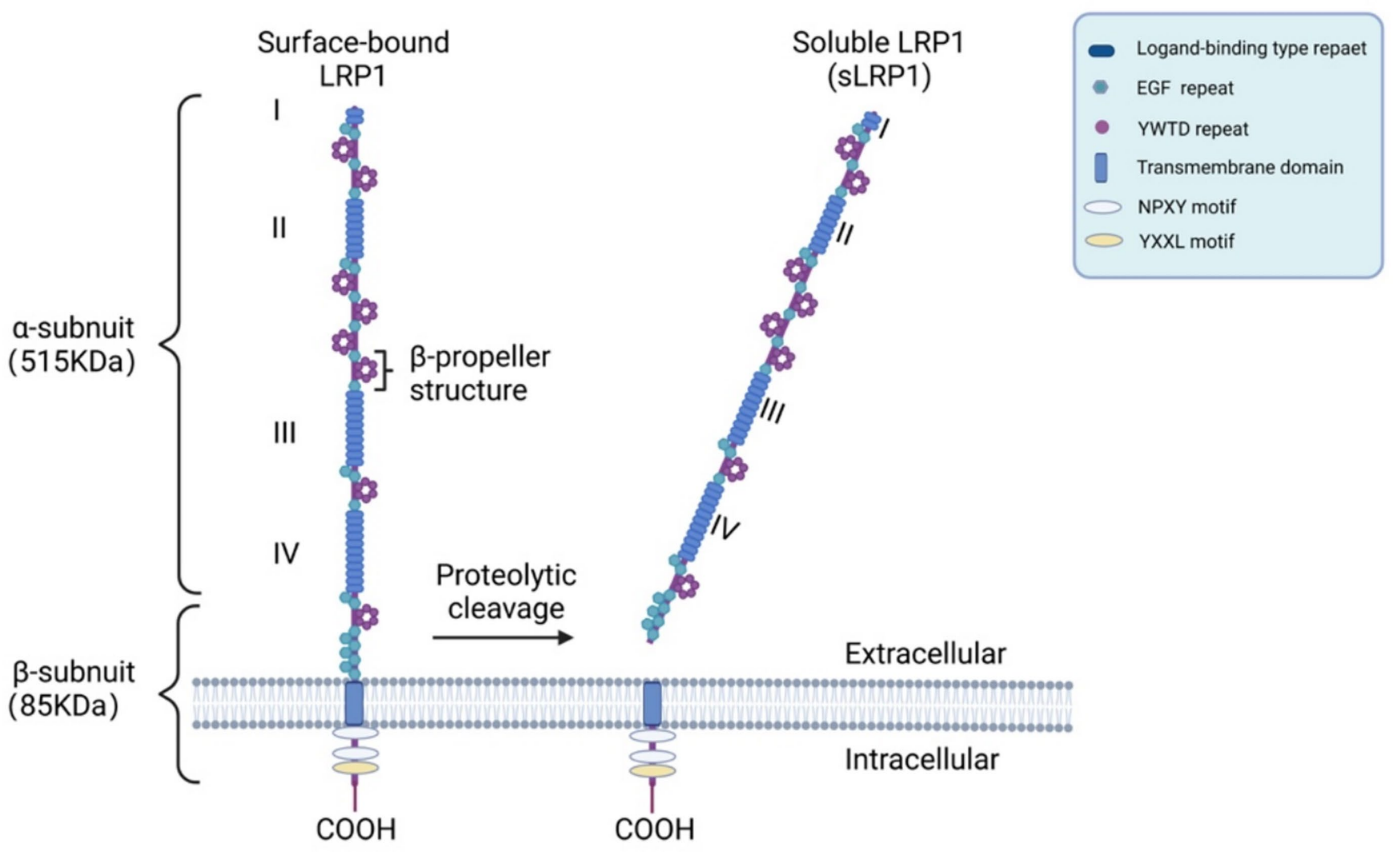
The structure of LRP1 and sLRP1. **(A)** LRP1 is characterized by one or more ligand-binding domains, epidermal growth factor (EGF), homology domains consisting of EGF repeats and YWTD propeller domains involved in pH-dependent release of ligands in the endosomes, a single transmembrane domain and a cytoplasmic tail containing at least one NPXY motif. **(B)** sLRP1 is produced by β-Secretase (BACE) proteolytic cleavage of the N-terminus extracellular domain of LRP1 (created by BioRender).

LRP1 was first identified in a screen for LDL receptor (LDLR) homologous sequences within the apoE domain in mouse lymphocytes and a human liver cDNA library (Herz et al., 1988). The LDLR family comprises major apoE receptors, including LDLR, LRP1, VLDLR and apoER2, each exhibiting different affinities for various apoE isoforms and lipidation states (Bu, 2009). Among these receptors, LRP1 is a type I transmembrane protein belonging to the LDLR family and is predominantly found in endothelial cells, vascular mural cells, neurons and astrocytes in neurovascular interface (Zlokovic, 2010; Sagare et al., 2012) (Figure 2). It consists of two subunits: a 515 kDa α-chain (ligand-binding domain) and an 85 kDa β-chain (transmembrane signaling domain), which are generated through *Furin*-mediated cleavage and remain associated via noncovalent interactions (Shinohara et al., 2017; Petralla et al., 2024). These two subunits work together to regulate ligand internalization and downstream signal transduction. Extracellularly, LRP1 contains four ligand-binding domains (I–IV), with domains II and IV being the primary binding sites (Neels et al., 1999; Obermoeller-McCormick et al., 2001). Additionally, its cytoplasmic tail includes two NPXY motifs and one YXXL motif, which play crucial roles in the receptor’s endocytic mechanisms (Li et al., 2000; Krieger and Herz, 1994).

## 3 Aβ transports across BBB

Studies have shown that Aβ protein is present not only in the brains of AD patients but also in the cerebrospinal fluid (CSF) and plasma of healthy individuals, with CSF Aβ levels being approximately 10 times higher than those in plasma (Kawarabayashi et al., 2001; Janelidze et al., 2016; Bateman et al., 2006). Recent research further confirms that Aβ accumulation in the brains of AD patients primarily results from impaired clearance mechanisms rather than simply an increased rate of Aβ production, which underscores the central role of the clearance system in AD pathogenesis (Bateman et al., 2006; Mawuenyega et al., 2010). Aβ is cleared from the brain through multiple pathways, including neuronal and glial uptake, transport across the BBB into circulation for systemic metabolism (e.g., in the liver and kidneys), enzymatic degradation (Iwata et al., 2004), bulk flow clearance via interstitial fluid (ISF) and absorption into CSF (Tarasoff-Conway et al., 2015; Hladky and Barrand, 2018). Studies have shown that LRP1-mediated clearance is significantly faster—up to six times more effective—than other pathways such as ISF flow or enzymatic degradation (Bell et al., 2007). Quantitative data indicate that LRP1 accounts for nearly 47.5% of Aβ1–42 clearance across the BBB and about 27% of total Aβ clearance, surpassing the efficiency of glymphatic and perivascular routes, which are slower and less specific (Storck et al., 2016). In addition, it is estimated that 40–60% of brain-derived Aβ is degraded through peripheral pathways outside the central nervous system (Cheng et al., 2020; Yuede et al., 2016). Among the key receptors involved in Aβ transport across brain cells and the BBB, LRP1 plays a pivotal role (Shinohara et al., 2017; Storck et al., 2016). Moreover, the affinity of Aβ protein for LRP1 is highly dependent on the aggregation state of Aβ (Wiatrak et al., 2021). Studies indicates that fibrillar and oligomeric Aβ forms bind to LRP1 with much higher affinity compared to the monomeric form (Petralla et al., 2024; Rushworth et al., 2013; Chen et al., 2017; Martiskainen et al., 2013).

Furthermore, LRP1 can undergo cleavage by α- and β-secretases, generating a soluble circulating form known as sLRP1 which is the primary binding receptor for circulating Aβ and binds approximately 70% of plasma Aβ₁₋₄₀ and 90% of Aβ₁₋₄₂ (Martiskainen et al., 2013; Liu et al., 2009; Sagare et al., 2007; Quinn et al., 1997). However, compared to healthy controls, sLRP1 levels are significantly reduced and the oxidized form of sLRP1 is elevated by 2.8–3.7 times in AD patients, which has a markedly lower affinity for Aβ and leads to a 30–35% reduction in Aβ binding (Sagare et al., 2007). Additionally, RAGE and P-gp are also critical for Aβ transport across the BBB (Yan et al., 1996; McCormick et al., 2021). RAGE is a multi-ligand cell surface receptor primarily expressed in brain endothelial cells, facilitating Aβ transport from the extracellular surface into brain (McCormick et al., 2021; Giri et al., 2000; Takuma et al., 2009). P-gp, an ATP-dependent efflux transporter, is predominantly expressed in neurons, glial cells and endothelial cells, where it directly interacts with Aβ and pumps it from endothelial cells into the bloodstream (McCormick et al., 2021; Storck et al., 2018; Hartz et al., 2010; Chai et al., 2022). Moreover, a portion of extracellular Aβ in the brain may diffuse passively across the BBB into the bloodstream via paracellular pathways (Keaney et al., 2015). In AD models, the expression levels of LRP1, P-gp and RAGE undergo significant changes, which may serve as initiating factors contributing to Aβ accumulation in the brain (Shibata et al., 2000; Alkhalifa et al., 2023).

## 4 The role of LRP1 in Aβ and tau metabolism

### 4.1 LRP1 influences Aβ production

Studies have shown that the cytoplasmic C-terminal domain of LRP1 interacts with the cytoplasmic domain of APP to influence Aβ production (Pietrzik et al., 2004; Waldron et al., 2008). It was observed that long-term cell culture in the presence of receptor-associated protein (RAP) led to an increase in cell surface APP levels and a significant reduction in Aβ synthesis (Ulery et al., 2000). Additionally, APP levels were reduced on the surface of Chinese hamster ovary (CHO) cells stably expressing LRP1, while APP accumulated on the surface of LRP1-deficient cells (Cam et al., 2005). In the brains of PDAPP mice, overexpression of LRP1 was found to increase soluble brain Aβ levels with age (Zerbinatti et al., 2004). One study demonstrated that LRP1 disruption *in vivo* shifted APP processing from amyloidogenic β-secretase cleavage, which generates Aβ, to non-amyloidogenic α-secretase cleavage, suggesting that LRP1 dysfunction might help reduce Aβ production (Van Gool et al., 2019). These findings indicate that LRP1 plays a role in APP processing and Aβ production. However, another study reported that in APP-transfected cells, LRP1 competed with APP for γ-secretase to reduce Aβ production (Lleó et al., 2005). Furthermore, conditional deletion of LRP1 in vascular smooth muscle cells led to impaired cerebrovascular Aβ clearance and cerebrovascular amyloid angiopathy (CAA)-like pathology, highlighting LRP1’s distinct role in Aβ drainage through the meningeal lymphatic system (Kanekiyo et al., 2012). These conflicting findings may be attributed to differences in cell types, experimental conditions and functional states of LRP1. Additionally, while some studies suggest that LRP1 overexpression increases Aβ levels, these results are primarily based on cell models. It has been well established that LRP1 expression declines with age in both human and mouse brains, with an even more pronounced reduction in AD (Deane et al., 2004; Kang et al., 2000; Donahue et al., 2006). It suggests that findings from LRP1 overexpression models may not directly relate to AD pathology. Therefore, further investigations using diverse AD models are needed to clarify whether LRP1 promotes Aβ production.

### 4.2 LRP1 and Aβ clearance

Studies have shown that downregulation of LRP1 leads to net accumulation of Aβ in the brain, which exacerbates neurodegeneration and creates a positive feedback loop in AD pathology by activating the pro-inflammatory phenotype of microglia (Jaeger et al., 2009). Recent research has found that the loss of phosphatidylinositol-binding clathrin assembly protein (PICALM) reduces Aβ clearance rates in mice and accelerates Aβ pathology (Storck et al., 2018; Zhao et al., 2015). Additionally, deletion of LRP1 in vascular smooth muscle cells disrupts lysosome-mediated Aβ clearance along the cerebral vasculature, leading to exacerbated Aβ deposition, which suggests that LRP1 plays a role in localized Aβ degradation (Kanekiyo et al., 2012). In primary mouse astrocytes, knock outing LRP1 not only impairs Aβ uptake and degradation but also downregulates Aβ-degrading enzymes, indicating that LRP1 may influence extracellular Aβ metabolism as well (Liu et al., 2017).

**Figure 3:**
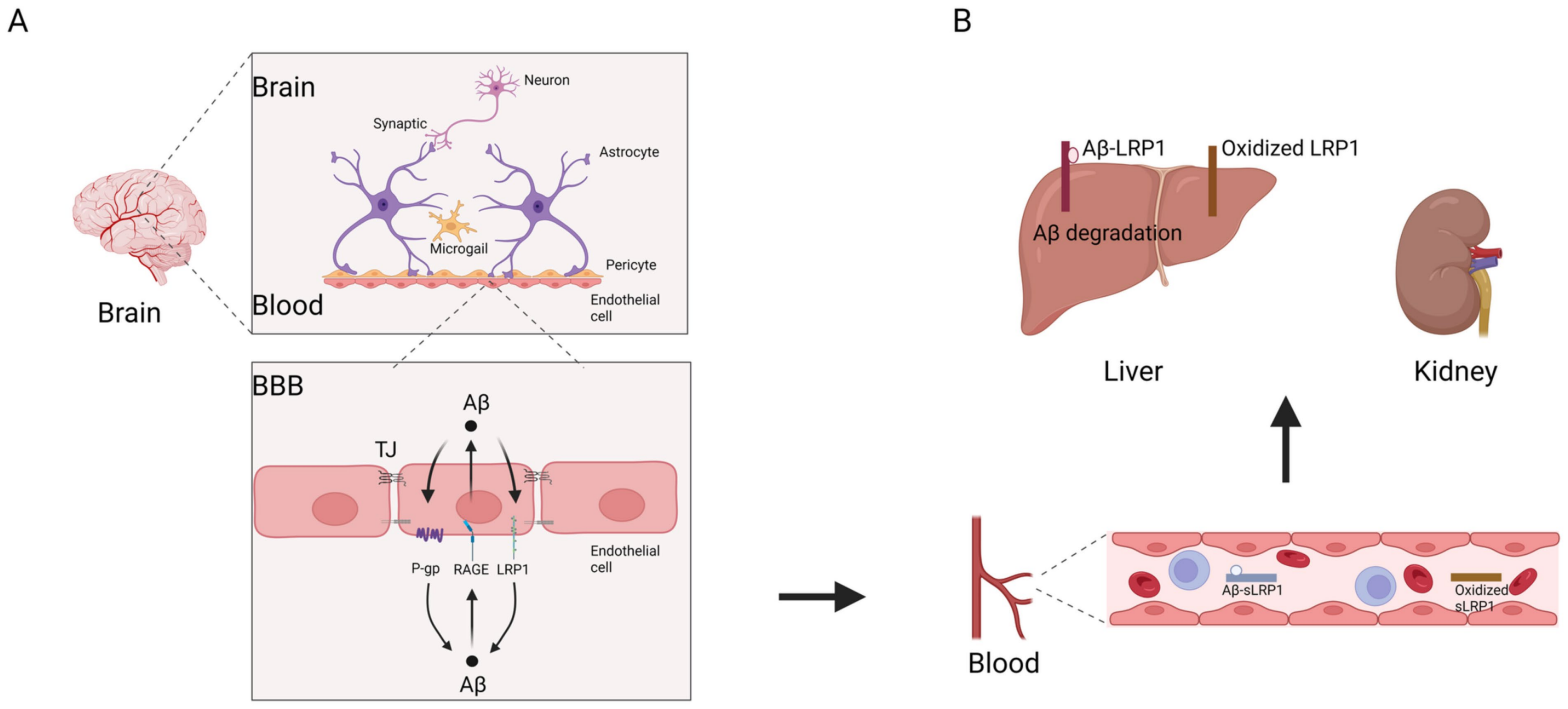
Schematic diagram illustrating the role of LRP1 and sLRP1 in a three-step process controlling clearance of brain Aβ. Step 1: Transcytosis of Aβ by LRP1 on the abluminal surface of brain endothelial cells, from brain to blood across the BBB. Step 2: Binding of plasma Aβ by sLRP1. Step 3: Clearance of systemic Aβ and sLRP1–Aβ complex by the liver and possibly the kidneys, in normal physiological conditions **(A)** and in AD **(B)**. Ligand binding domains II and IV (green) bind Aβ (round orange). Oxidized (red) LRP1 or sLRP1 does not bind Aβ. (created by BioRender).

LRP1 is highly expressed in multiple human organs, including the liver, brain, lungs, intestines and vascular system (Moestrup et al., 1992). When blood circulates through the liver, LRP1 clears 13.9% of Aβ₄₂ and 8.9% of Aβ₄₀ by mediating receptor endocytosis and lysosomal degradation, with Aβ metabolites being excreted into the intestines via bile ducts (Cheng et al., 2023; Tamaki et al., 2006; Ghiso et al., 2004) (Figure 3). Meanwhile, the kidneys primarily eliminate Aβ through glomerular filtration, and together, these organs help maintain peripheral Aβ homeostasis (Ghiso et al., 2004; Hone et al., 2003). According to the “peripheral sink” hypothesis, a dynamic equilibrium exists between peripheral and brain Aβ pools, where upregulating hepatic LRP1 levels enhances peripheral Aβ clearance, facilitating the removal of brain Aβ into the bloodstream via the BBB (DeMattos et al., 2001; Matsuoka et al., 2003; Vasilevko et al., 2007). Notably, plasma Aβ levels increase with age, suggesting an age-dependent decline in hepatic Aβ uptake (Smith and Betteridge, 2004). This indicates that liver dysfunction may contribute to Aβ accumulation in the brain and accelerate AD progression (Wang Y. R. et al., 2017; Peng et al., 2024; Wang J. et al., 2017). LRP1 not only serves as the primary receptor for hepatic Aβ uptake from circulation but also functions as an Aβ clearance receptor in liver (Tamaki et al., 2006; Kanekiyo and Bu, 2014; Mohamed and Kaddoumi, 2013). Liver-specific overexpression of LRP1 has been shown to reduce brain Aβ burden by enhancing peripheral Aβ sequestration which also rescues synaptic plasticity deficits and spatial memory impairments in APP/PS1 mice, supporting the therapeutic potential of the peripheral sink hypothesis (Sehgal et al., 2012). Given LRP1’s pivotal role in Aβ clearance, developing therapeutic strategies to target LRP1 may offers promising avenues for AD treatment.

### 4.3 The role of LRP1 in tau pathology

Studies have shown that knocking out LRP1 significantly reduces tau uptake in glioblastoma cells and pluripotent stem cell-derived neurons. *In vivo*, downregulation of LRP1 effectively limits tau propagation between neurons in a tau-spreading mouse model (Rauch et al., 2020). However, given LRP1’s crucial role in Aβ clearance, inhibiting LRP1 to reduce tau spread may simultaneously impair Aβ clearance, necessitating careful evaluation of its therapeutic potential (Eisenbaum et al., 2024). Research indicates that the addition of oligomeric tau (TauO) to C6 cells downregulates LRP1 levels, while LRP1 expression is upregulated in heparan sulfate proteoglycan (HSPG)-deficient cells, which suggests that surface HSPG influences LRP1-mediated tau uptake (Song et al., 2022). Targeting HSPG may therefore provide a strategy to reduce tau internalization LRP1 also binds and processes monomeric tau, facilitating its degradation and promoting the seeding of pathological tau (Cooper et al., 2021). Recent findings further suggest that TauO upregulates mitochondrial NADPH synthesis via NADK2, which affects LRP1-mediated TauO internalization (Pardo et al., 2024). Additionally, LRP1 may participate in the gut-brain axis by mediating the trans-barrier transport of tau fibrils, highlighting the potential for gut microbiome modulation or intestine-specific LRP1 inhibition as a novel strategy to block tau pathology spread (Ahn et al., 2024). Despite these findings, blocking LRP1 function to prevent tau propagation in the brain may not yield therapeutic benefits, as any potential advantages could be counteracted by network dysfunction and increased amyloid deposition (Deinhardt, 2020). Thus, LRP1-targeted interventions require a nuanced approach to balance its effects on both tau and Aβ pathologies.

### 4.4 LRP1’s other metabolic functions

Beyond its role in Aβ and tau metabolism, LRP1 has multiple other functions. After its intracellular domain translocate to the nucleus, LRP1 can regulate gene transcription and expression, as well as serve as a scaffold for intracellular adaptor proteins (Auderset et al., 2016; Potere et al., 2019). LRP1 also modulates various signaling pathways by interacting with platelet-derived growth factor (PDGF) (Muratoglu et al., 2010; Craig et al., 2013), N-methyl-D-aspartate (NMDA) receptors (May et al., 2004; Maier et al., 2013) and the leptin/leptin receptor complex in the hypothalamus (Marwarha and Ghribi, 2012). Additionally, LRP1 plays a role in insulin signaling and glucose metabolism by interacting with insulin receptors and reducing the levels of glucose transporters GLUT3 and GLUT4 in neurons (Liu et al., 2015; Gali et al., 2019). Intravenous injection of an IGF1R/IR kinase inhibitor in wild-type mice significantly reduces LRP1 levels, highlighting its importance in insulin signaling (Zhou et al., 2024). In diabetes, high glucose environments and experimental diabetic models reduce LRP1 expression and function at the BBB, lowering Aβ clearance and worsening cognitive impairment (Yavari et al., 2025; Xue et al., 2022). In neurodegenerative diseases, LRP1 serves as a key regulator of α-synuclein uptake by neurons and is a crucial mediator of its spread in Parkinson’s disease (Chen et al., 2022). These findings underscore the diverse metabolic functions of LRP1, suggesting that its dysregulation may contribute to various pathological conditions and disease outcomes.

## 5 Factors affecting LRP1 function

### 5.1 Metabolic influence of APOE

As a key receptor *in vivo*, LRP1 function is influenced by multiple factors, where APOE is an essential one. LRP1-mediated Aβ transport across the BBB is regulated by APOE (Zhang et al., 2022; Bell et al., 2007). Studies found that diet-related changes (notably high-fat diets) can further disturb the gut-brain-lipid axis, affecting BBB permeability and the expression/function of key proteins such as LRP1 and APOE (Luo et al., 2024). The polymorphic variants of APOE (ε2/ε3/ε4) significantly influence AD risk by modulating the stability and clearance efficiency of the Aβ-LRP1 complex (Huang and Mahley, 2014). Gut microbiota composition and their metabolites (such as short-chain fatty acids and secondary bile acids) modulate APOE expression and its neuroprotective or pathological effect, such as APOE2 carriers often have a gut microbiome that supports neuroprotection through higher levels of beneficial metabolites (Luo et al., 2024; Corder et al., 1994; Talbot et al., 1994), while APOE4 is linked to reduced neuroprotection, perturbed cholesterol homeostasis, and increased Aβ pathology (Huang and Mahley, 2014; Mah et al., 2023; Corder et al., 1993; Gokulan et al., 2025). In EOAD patients carrying the APOE ε4 allele, CSF levels of phosphorylated tau181 (P-tau181) are higher than in LOAD patients (Lv et al., 2023). This could be one of the reasons why EOAD patients exhibit more severe memory and cognitive decline (Falgàs et al., 2024). Additionally, the ε4 allele differentially affects the relationship between tau and Aβ in EOAD and LOAD (Kang et al., 2023). As the most significant genetic risk factor for late-onset AD, APOE ε4 increases AD risk in the number of APOE ε4 alleles and lowers the age of onset (Mah et al., 2023; Corder et al., 1993). APOE4 also promotes early Aβ seeding through its neuron-specific interaction with LRP1 (Strickland and Holtzman, 2019). However, when APOE ε4 expression is reduced in ε4 carriers, overall APOE levels will increase and lead to lower Aβ accumulation in the brain (Kanekiyo et al., 2014).

**Figure 4:**
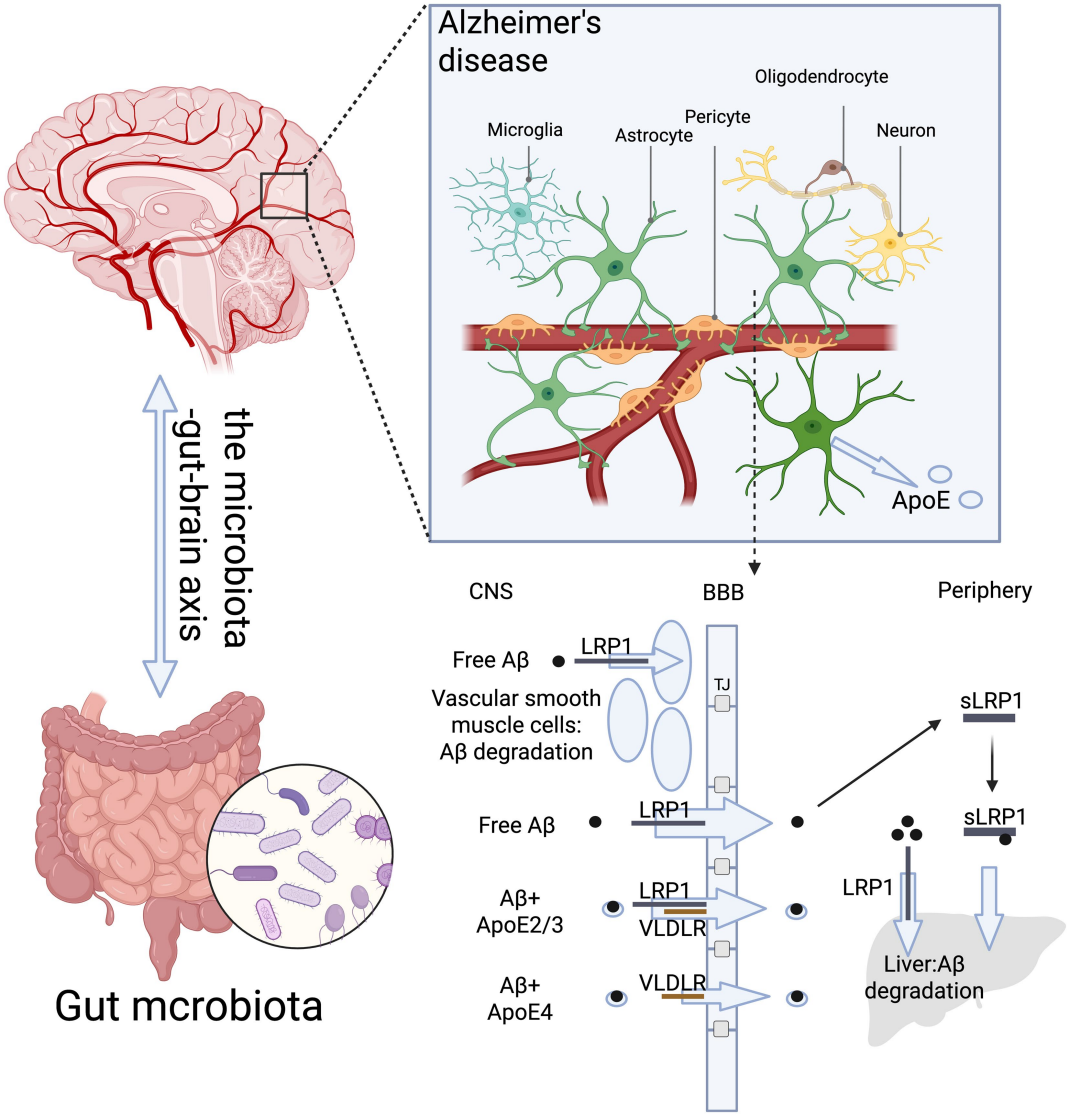
APOE mediates Aβ-LRP1 complexes across the BBB. The gastrointestinal-brain axis (GBA) modulates the risk and progression of AD by integrating gut/liver-derived signals, systemic metabolism, and neuroinflammation, altering the function of APOE and LRP1 in lipid transport and Aβ clearance. APOE4-Aβ complex is cleared not by LRP1 but by very-low-density lipoprotein receptor (VLDLR), while APOE2-Aβ and APOE3-Aβ complexes are cleared by both LRP1 and VLDLR at a faster internalization rate than APOE4-Aβ (created by BioRender).

APOE expression plays a crucial role in the endocytosis and transcytosis of Aβ-LRP1 complexes across the BBB. For example, studies show that human APOE transgenic mice clear Aβ more efficiently than APOE-knockout or wild-type mice (Bachmeier et al., 2013). Moreover, compared to free Aβ, APOE-bound Aβ has a reduced clearance rate at the BBB in mice (Deane et al., 2008). APOE isoforms directly interact with LRP1 and may compete with Aβ for cellular clearance, thereby affecting brain Aβ clearance differently (Verghese et al., 2013). Compared to apoE2- and apoE3-expressing mice, apoE4-expressing mice exhibit increased hippocampal Aβ deposition in APP/PS1 models (Tachibana et al., 2019). In addition, APOE2 carriers often have a gut microbiome that supports neuroprotection through higher levels of beneficial metabolites, while APOE4 is linked to reduced neuroprotection, perturbed cholesterol homeostasis (Luo et al., 2024). Dysbiosis can increase systemic inflammation and intestinal permeability and activation of inflammatory pathways (especially in APOE4 carriers) impairs LRP1-mediated Aβ clearance (Erickson et al., 2012; Kang and Wang 2024). Research indicates that the APOE4-Aβ complex is cleared not by LRP1 but by very-low-density lipoprotein receptor (VLDLR), while APOE2-Aβ and APOE3-Aβ complexes are cleared by both LRP1 and VLDLR at a faster internalization rate than APOE4-Aβ (Deane et al., 2008) (Figure 4). Additionally, studies show that LRP1 exacerbates Aβ pathology in APOE ε4 carriers, while this effect is not observed in non-carriers and knocking out neuronal LRP1 will prevent APOE4-induced Aβ pathology (Strickland and Holtzman, 2019; Tachibana et al., 2019). However, such a therapeutic approach might have severe pathological consequences, requiring further investigation.

### 5.2 Genetic influence factors

LRP1 expression is also regulated by genetic factors. The transcriptional repressor SREBP2 negatively regulates LRP1 expression (Llorente-Cortés et al., 2006; Llorente-Cortés et al., 2007). Genes involved in vascular and metabolic functions can influence Aβ clearance in the central nervous system through SREBP2 (Moon et al., 2011; Fan et al., 2019). For instance, defects in the SLC2A1 gene, which encodes GLUT1, lead to upregulation of the SREBP2 transcription factor, thereby reducing LRP1 expression (Winkler et al., 2015). Additionally, overexpression of SRF/MYOCD in cerebral endothelial cells of AD patients significantly decreases LRP1 levels compared to age-matched healthy controls (Bell et al., 2009). MEOX2, a regulator of vascular differentiation and remodeling, is downregulated in AD and Low levels of MEOX2 promote proteasomal degradation of LRP1 to reduce its expression (Wu et al., 2005). However, another study reported that MEOX2 haploinsufficiency does not affect Aβ plaque deposition in APP/PS1 mice (Soto et al., 2016). The contradictory findings may be due to differences in study models, requiring further investigation into MEOX2’s impact on LRP1 expression. In summary, LRP1 function and expression are regulated by multiple factors, some of which may provide new therapeutic targets for AD.

### 5.3 Other influence factors

Age-dependent decline in LRP1 expression may be a key molecular basis for the high prevalence of LOAD (Shibata et al., 2000). Additionally, various proteases, including β-secretase1 (BACE-1) (von Arnim et al., 2005), membrane-type 1 matrix metalloproteinase (MT-1MMP) (Rozanov et al., 2004), ADAM family metalloproteinases (ADAM10, ADAM17) (Liu et al., 2009) and tissue plasminogen activator (t-PA) (Polavarapu et al., 2007), can cleave LRP1 from the cell surface, increasing sLRP1 levels in the bloodstream (Quinn et al., 1997). Oxidative stress disrupts the ability of LRP1 and sLRP1 to bind Aβ and Aβ induces LRP1 oxidation in turn, further impairing its ability to clear Aβ (Ramanathan et al., 2015; Beiyu et al., 2024). Pro-inflammatory cytokines such as IL-1β, IL-6, and TNF-α downregulate LRP1 expression in human MVECs and BLECs to promote Aβ accumulation, which also highlighting the role of neuroinflammation in AD progression (Hsu et al., 2021; Versele et al., 2022). Certain metals also impact LRP1 function, for example, aluminum downregulates LRP1 levels in PC12 cells, while lead exposure reduces LRP1 expression in cortical capillaries and the hippocampus (Liu et al., 2023; Wu S. et al., 2020; Zhang et al., 2019; Shen et al., 2020). High blood glucose and cholesterol levels lower LRP1 expression in brain microvascular endothelial cells, affecting Aβ efflux from the brain (Xue et al., 2022; Zhou et al., 2021). Additionally, chronic alcohol consumption decreases hepatic LRP1 expression and increases brain Aβ levels (Chandrashekar et al., 2024; Garcia et al., 2022).

## 6 Changes in LRP1 expression levels in AD patients

LRP1 is highly expressed in neurons, particularly in key brain regions associated with AD, such as the cerebral cortex, hippocampus and striatum (Li et al., 2025). Studies indicate that compared to non-dementia controls (NDC), LRP1 mRNA expression is elevated in brain regions like the hippocampus and temporal cortex (Qiu et al., 2001). However, LRP1 expression in AD patients exhibits spatial heterogeneity: vascular endothelial LRP1 is compensatory upregulated in response to increased Aβ burden, while neuronal LRP1 is downregulated, potentially exacerbating synaptic Aβ toxicity and tau aggregation (Donahue et al., 2006; Jeynes and Provias, 2008). In addition, decreased LRP1 mRNA levels have been observed in the cerebellum, middle frontal cortex and occipital lobe of AD patients (Storelli et al., 2021; Ruzali et al., 2012). Moreover, AD patients show a significant decline in plasma sLRP1 levels, along with increased oxidative modifications, which may be related to reduced hepatic LRP1 synthesis and increased MMP-9-mediated shedding (Gao et al., 2020). Overall, the significant alterations in LRP1 expression across different brain regions in AD suggest that regulating LRP1 levels in these areas could be a potential therapeutic strategy for AD.

## 7 The translational potential of LRP1 as an early diagnostic biomarker

As a primary receptor for Aβ binding, LRP1 loss in endothelial cells is observed not only in aging populations but also in the early stages of AD (Kang et al., 2000). Evidence suggests that plasma sLRP1 levels are significantly lower in APOE ε4 carriers compared to non-carriers, with a negative correlation between APOE ε4 status and plasma sLRP1 levels (Jiang et al., 2022; Wang et al., 2023). Additionally, in mild cognitive impairment (MCI) patients, oxidative modifications of sLRP1 impair its ability to bind Aβ, which correlates with cognitive decline as MCI progresses to AD (Sagare et al., 2011). Another study reported that the sensitivity of sLRP1 for AD diagnosis reached 77.8%and combining sLRP1 with soluble receptor for advanced glycation end-products (sRAGE) improved diagnostic accuracy compared to using either biomarker alone (Liang et al., 2013). These findings indicate that sLRP1 holds promise as an early biomarker for MCI-to-AD conversion, though further validation is required to establish its diagnostic efficacy. The precise quantification of LRP1 and sLRP1 is crucial for advancing this research area. The most used method, ELISA, has limitations such as potential cross-reactivity and the inability to provide molecular size information. To address these challenges, researchers have explored liquid chromatography–tandem mass spectrometry (LC–MS/MS) for detecting LRP1 levels in vitreous and corneal tissues to achieve high sensitivity and specificity (Velez et al., 2023; Mogensen et al., 2022). However, LC–MS/MS is complex and costly, limiting its widespread use. Developing more efficient and accessible LRP1 detection methods is essential to fully realize its potential as an early diagnostic biomarker for AD patients.

## 8 LRP1 as a therapeutic target for Alzheimer’s disease

Given the critical role of LRP1 in Aβ metabolism, developing targeted therapies to regulate LRP1 activity and expression holds significant clinical potential for AD. Multiple studies have investigated whether modulating LRP1 expression can promote Aβ clearance and slow AD progression. Current research on LRP1-targeted therapies primarily focuses on pharmacological and genetic approaches. Existing therapeutic strategies aim for dual regulation: one is Enhancing membrane-bound LRP1 activity in the BBB and liver to facilitate Aβ transport and clearance, another is stabilizing or supplementing circulating sLRP1 to maintain peripheral Aβ-binding capacity to restore the pathological imbalance in brain-periphery Aβ dynamics.

### 8.1 Pharmacological therapy

Studies have found that tissue-nonspecific alkaline phosphatase (TNAP), an ecto-enzyme upregulated in the brains of AD patients, inhibits LRP1-mediated Aβ transport across the BBB and Block TNAP can enhance Aβ clearance through LRP1-mediated transport across the BBB (Soria-Tobar et al., 2024). Granule calcium protein (GCA) competes with Aβ for LRP1 binding in microglia and treatment with GCA-neutralizing antibodies has been shown to increase LRP1 levels and inhibit AD progression (Zhou et al., 2023). A recent study also found that exposure to low-intensity blasts may enhance endothelial clearance of Aβ through LRP1-mediated transcytosis (Abutarboush et al., 2024). It suggests that future research may explore using sound and pressure as potential therapeutic approaches for AD. These findings represent novel and potentially effective treatment strategies for AD patients, though further investigation is required.

Significant progress has been made in studying pharmacological approaches to regulate LRP1 levels and promote Aβ clearance. Studies have demonstrated that cholesterol-lowering statins can enhance LRP1 expression and promote Aβ clearance. For instance, fluvastatin reduces Aβ levels by increasing LRP1 expression in cerebral microvessels (Shinohara et al., 2010). Simvastatin has been found to upregulate LRP1 expression in human brain microvascular endothelial cells (HBMECs) (Zandl-Lang et al., 2018). Moreover, Angiopep-2-modified statin nanoparticles can penetrate the BBB via LRP1-mediated receptor transcytosis while simultaneously inducing endothelial LRP1 upregulation, leading to both Aβ clearance and neuroprotection (Guo et al., 2019). Additionally, thiazolidinedione drugs such as low-dose rosiglitazone enhance Aβ clearance by upregulating LRP1 mRNA and protein expression in HBMECs and increasing LRP1 promoter activity (Moon et al., 2012). Low-dose pioglitazone similarly enhances Aβ clearance by increasing LRP1 activity (Seok et al., 2019).

Moreover, lithium can enhance LRP1 transcription by inhibiting GSK-3β, while magnesium regulates metal ion homeostasis to maintain LRP1 structural activity and a ketogenic diet activates the PPARγ-LRP1 axis through β-hydroxybutyrate, optimizing Aβ brain–blood distribution via multiple pathways (Versele et al., 2020; Pan et al., 2018; Zhu et al., 2018). Supplementation with omega-3 polyunsaturated fatty acids (PUFAs) has also been shown to increase LRP1 expression in HBMECs (Yan et al., 2020). Studies have revealed that canolol can activate the Nrf2/ARE pathway to reduce oxidative stress at the BBB while upregulating both P-gp and LRP1 expression to enhance Aβ efflux and degradation (Qosa et al., 2015). Andrographolide, a bioactive component of *Andrographis paniculata*, promotes cell viability and increases LRP1 expression while reducing neuroinflammation to mitigate Aβ-induced cytotoxicity (Ju et al., 2023). The traditional Chinese medicine formula ZhengXingshuiYizhi Fang (XSF) has been found to inhibit Aβ-induced endothelial apoptosis via the PI3K/Akt/NF-κB pathway and restore Aβ clearance across the BBB by upregulating LRP1 expression, highlighting the potential of multi-target interventions in AD treatment (Wu L. et al., 2020).

In cultured murine brain endothelial cells, treatments with Lycopene Aldehyde or canolol increased P-gp and LRP1 expression and activity, with canolol also exhibiting notable anti-inflammatory effects (Qosa et al., 2015; Abuznait et al., 2013). Moderate ethanol exposure (MEE) was found to reduce neuroinflammation and Aβ deposition in APP/PS1 mice by regulating LRP1 expression (Kang et al., 2024). Tanshinone IIA (Tan IIA) enhances LRP1 expression in both animal models and cultured cells, promoting Aβ transport by alleviating SIRT1-mediated endoplasmic reticulum (ER) stress in brain microvascular endothelial cells (BMECs) to improve cognitive deficits in APP/PS1 mice (Wan et al., 2023). Gardenia-derived flavonoid GJ-4 has been reported to increase LRP1 expression and significantly improve spatial learning and memory abilities in AD mouse models (Zhang et al., 2020). *In vitro* studies using bexarotene (Bex) and astaxanthin (Asx) on 3xTgAD mice have demonstrated that Bex and Asx can reduce BACE1 expression while increasing LRP1 expression in microvascular brain endothelial cells and enhance Aβ clearance (Fanaee-Danesh et al., 2019). Similarly, research on the methanol extract of *Moringa oleifera* (MO) in APP/PS1 mice has shown that MO treatment increases LRP1 expression while reducing BACE1 and other protein levels, effectively lowering Aβ burden to levels comparable to wild-type control mice (Mahaman et al., 2022).

In the liver, the cholesterol-lowering drug atorvastatin has been shown to promote peripheral Aβ clearance by inducing SREBP2, which upregulates hepatic LRP1 levels (Moon et al., 2011). Additionally, extracting from *withania somnifera* (Ashwagandha) not only increases LRP1 expression in the liver but also elevates sLRP1 levels in the bloodstream and facilitates Aβ metabolism (Sehgal et al., 2012). In addition, replacing oxidized sLRP1 to restore Aβ binding and transport in plasma represents a promising therapeutic strategy for AD.

However, merely increasing LRP1 levels may not be sufficient. For example, Aβ clearance remains impaired despite increased LRP1 expression in the brain of mice lacking vitamin E synthase (Nishida et al., 2009). It suggests that additional cofactors may be involved in Aβ clearance processes. Moreover, overexpression of LRP1 may have potential drawbacks, as it has been implicated in promoting APP amyloid genesis and facilitating amyloid accumulation in neurons. Additionally, drugs that regulate LRP1 levels may affect the clearance of other molecules and metabolites, potentially disrupting broader metabolic processes. Thus, further research is necessary to fully understand the therapeutic implications of LRP1-targeted pharmacological interventions for AD treatment.

### 8.2 Gene therapy

Gene therapy, as an emerging treatment approach for AD, aims to correct abnormal gene expression by introducing specific genes into patient cells. Studies have found that the absence of ANKS1A protein in endothelial cells leads to reduce surface LRP1 levels and decrease Aβ clearance across the BBB. Brain endothelial-specific ANKS1A gene therapy can reverse these defects by restoring LRP1 levels and facilitating Aβ clearance through the BBB (Lee et al., 2023). Another study identified NYGGF4 as a novel LRP1-interacting protein that specifically binds to LRP1. Compared to age-matched control groups, NYGGF4 expression in AD patients gradually decreases alongside LRP1 as the disease progresses (Kajiwara et al., 2010). It suggests that targeting NYGGF4-LRP1 interactions may provide a potential strategy for upregulating LRP1 expression in AD treatment. In past research, adeno-associated virus (AAV) has been used as a gene therapy vector, enabling long-term gene expression in both dividing and non-dividing cells (Grieger and Samulski, 2012). One study revealed that reduced mNAT1 expression leads to a decline in regulators responsible for exporting Aβ-LRP1 complexes and researchers successfully reinstated the normal export function of Aβ-LRP1 complexes by selectively restoring mNAT1 expression in brain endothelial cells via AAV to reduce Aβ deposition (Zou et al., 2020).

Although these gene therapy strategies are still in the research phase and remain distant from clinical application, they hold distinct advantages over pharmacological treatments by minimizing drug-related toxicity risks and paving the way for personalized therapy for AD patients.

## 9 Conclusion and outlook

Current research has established LRP1 as a key player in Aβ clearance and metabolism, significantly influencing AD pathology. Targeting LRP1 as a therapeutic approach for AD, including pharmacological and gene therapies, is actively under development. Despite there is significant progress in understanding LRP1 expression and function, the clinical translation of LRP1-targeted therapy faces several challenges. One major issue is the dual role of LRP1, as it is involved in both Aβ clearance and APP processing. Developing subtype-selective regulatory tools, such as monoclonal antibodies targeting extracellular ligand-binding domains, could help overcome this limitation. As a central node in the Aβ metabolic network, LRP1 function is finely regulated by APOE isoforms, inflammatory microenvironments and epigenetic modifications. Future studies should leverage single cell sequencing and spatial transcriptomics to map the spatiotemporal dynamics of LRP1 activity. Additionally, since LRP1 participates in multiple physiological systems, the development of LRP1-based AD therapies requires rigorous toxicity and safety assessments to mitigate potential adverse effects. LRP1 expression and function are influenced by various factors, including inflammation, oxidative stress, receptor shedding and genetic signaling. Thus, overcoming off-target risks associated with its multifunctionality is crucial. Potential strategies include developing tissue-specific delivery systems (e.g., optimized AAV serotypes) and subdomain-selective LRP1 modulators to balance therapeutic efficacy and safety. Furthermore, whether LRP1-targeted therapy affects other neurological functions remains to be fully investigated. Future research should integrate LRP1 gene-editing models, organoid technology and *in vivo* imaging to elucidate its functional heterogeneity across different brain regions and cell types. Additionally, combining LRP1 modulators with anti-tau antibodies or neuroinflammation inhibitors could provide synergistic therapeutic benefits.

In conclusion, LRP1 plays a crucial role in AD pathogenesis and represents a promising therapeutic target. However, challenges such as its multifaceted functions and off-target effects must be addressed. Moving forward, the development of selective regulatory tools, the integration of advanced technologies and stringent safety monitoring will be essential to balance efficacy and safety, ultimately paving the way for clinical application.
